# “Ménage à Trois”: The Evolutionary Interplay between JSRV, enJSRVs and Domestic Sheep

**DOI:** 10.3390/v6124926

**Published:** 2014-12-09

**Authors:** Alessia Armezzani, Mariana Varela, Thomas E. Spencer, Massimo Palmarini, Frédérick Arnaud

**Affiliations:** 1 MRC-University of Glasgow Centre for Virus Research, 464 Bearsden Road, Glasgow G61-1QH, UK; E-Mails: alessia.armezzani@gmail.com (A.A.); mariana.varela@glasgow.ac.uk (M.V.); massimo.palmarini@glasgow.ac.uk (M.P.); 2 UMR5667, Laboratory of Plant Development and Reproduction, Institut National de la Recherche Agronomique, Ecole Normale Supérieure de Lyon, 46 Allée d’Italie, 69007 Lyon, France; 3 Department of Animal Sciences and Center for Reproductive Biology, Washington State University, PO Box 646310 Pullman, Washington, DC, USA; E-Mail: thomas.spencer@wsu.edu; 4 UMR754, Université Claude Bernard Lyon 1, Institut National de la Recherche Agronomique, Ecole Pratique des Hautes Etudes, SFR BioSciences Gerland, 50 avenue Tony Garnier, 69007 Lyon, France

**Keywords:** JSRV, enJSRV, retrovirus, virus-host co-evolution, restriction factors, placenta

## Abstract

Sheep betaretroviruses represent a fascinating model to study the complex evolutionary interplay between host and pathogen in natural settings. In infected sheep, the exogenous and pathogenic Jaagsiekte sheep retrovirus (JSRV) coexists with a variety of highly related endogenous JSRVs, referred to as enJSRVs. During evolution, some of them were co-opted by the host as they fulfilled important biological functions, including placental development and protection against related exogenous retroviruses. In particular, two enJSRV loci, enJS56A1 and enJSRV-20, were positively selected during sheep domestication due to their ability to interfere with the replication of related competent retroviruses. Interestingly, viruses escaping these transdominant enJSRVs have recently emerged, probably less than 200 years ago. Overall, these findings suggest that in sheep the process of endogenization is still ongoing and, therefore, the evolutionary interplay between endogenous and exogenous sheep betaretroviruses and their host has not yet reached an equilibrium.

## 1. Introduction

Retroviruses possess a unique replication cycle that requires the integration of viral DNA into the host genome to form a provirus. Normally, retroviruses integrate into somatic cells and are transmitted horizontally from infected to uninfected hosts as “exogenous” elements. However, occasionally, they can infect germ line cells and become permanent elements of the genome that are transmitted vertically to offspring as any other Mendelian gene; such inherited proviruses are known as “endogenous” retroviruses (ERVs) [1]. ERVs are found in all vertebrates studied to date, where they represent a significant percentage of the total genome: up to 8%–10% of human and mouse genomic DNA is thought to have retroviral origins [2].

ERVs are classified as “ancient” or “modern” depending on whether they integrated into the germ line before or after host speciation. Ancient ERVs invaded host genomes before speciation and are therefore located on the same chromosome in phylogenetically related species. However, most of them are inactivated due to the accumulation of nonsense mutations and/or genetic deletions over time. Interestingly, the majority of ancient ERVs do not possess any exogenous counterpart, reinforcing the hypothesis that the endogenization process partly contributes to the disappearance of exogenous infectious retroviruses [3]. Modern ERVs, on the other hand, integrated into the host DNA after speciation and exist both as endogenous and exogenous retroviruses. As such, they are usually not completely fixed in the host genome, but are present as insertionally polymorphic loci,* i.e.*, they are found only in some individuals or populations of their host species. Moreover, some modern ERVs possess intact open reading frames for most of their genes. Thus, they are potentially able to produce infectious particles and, in turn, can re-infect the host germ line and give rise to multiple ERVs copies within the host DNA [3]. Examples of modern ERVs are koala retroviruses, cervid endogenous gammaretroviruses (CrERVγs), porcine endogenous retroviruses (PERVs), and endogenous Jaagsiekte sheep retroviruses (enJSRVs) that are currently invading koala, mule deer, pigs, and sheep genomes, respectively, suggesting that in these animal species the process of endogenization is still taking place [4,5,6,7,8].

This review will trace the long-standing interplay between endogenous, exogenous sheep betaretroviruses and their host, with particular emphasis on the mechanisms and biological consequences of enJSRVs endogenization, their potential contribution to host evolution and biology, and their use for reconstructing their natural history and the one of their hosts.

## 2. Jaagsiekte Sheep Retrovirus (JSRV)

Jaagsiekte sheep retrovirus (JSRV) is the causative agent of ovine pulmonary adenocarcinoma (OPA), a naturally occurring lung cancer of sheep [9]. OPA is a common disease of sheep throughout the world, but is rarely found in goats and wild moufflons [10,11]. Importantly, it shares several characteristics with some forms of human lung adenocarcinomas, which makes it a fitting large animal model for lung carcinogenesis [12,13]. JSRV is unique among oncogenic retroviruses, since the expression of its envelope glycoprotein (Env) alone is sufficient to transform a variety of cell lines* in vitro*. More importantly, the JSRV Env *per se* is able to induce lung adenocarcinomas* in vivo* in mice and in immunocompetent sheep inoculated with a JSRV defective virus which expresses *env* under the control of its own long terminal repeat (LTR) [14,15].

**Figure 1:**
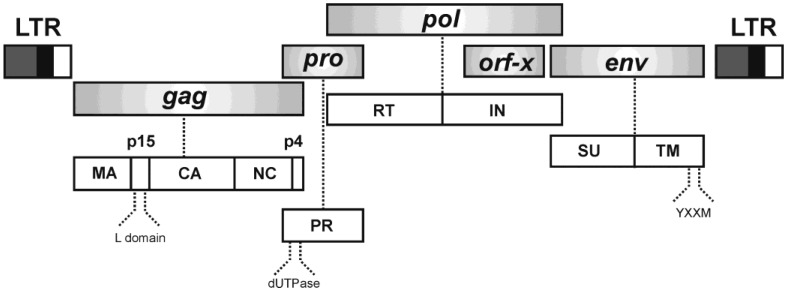
JSRV genomic organization. The JSRV genome encodes *gag*,* pro*,* pol* and *env* genes and *orf-x*, an additional open reading frame of unknown function (top). On the bottom are indicated the polyprotein precursors derived from each gene that are cleaved by the cellular machinery. See text for more details. LTR, long terminal repeat; MA, matrix; p15, 15 kDa protein; L domain, late domain; CA, capsid; NC, nucleocapsid; p4, 4 kDa protein; PR, protease; dUTPase, deoxyuridine triphosphatase; RT, reverse transcriptase; IN, integrase; SU, surface domain; TM, transmembrane domain; YXXM, Y indicates a tyrosine residue, X any amino acid and M a methionine residue (according to the single-letter code).

JSRV is a Betaretrovirus phylogenetically related to enzootic nasal tumor virus (ENTV), Mason-Pfizer monkey virus (M-PMV) and mouse mammary tumor virus (MMTV). To date, three highly related JSRV isolates have been cloned from OPA affected sheep: a South African strain of JSRV and two strains derived from United Kingdom, JSRV_21_ and JSRV_JS7_, with JSRV_21_ being the most thoroughly studied [9,16,17,18,19]. The genome of JSRV is approximately 7.5 kb in length and, besides encoding the classical retroviral genes *gag*, *pro*, *pol* and *env*, it harbors an additional open reading frame expressed from two singly spliced subgenomic mRNAs of 3.2 kb, and encoding a putative protein of 179 amino acids of unknown function (hence termed *orf-x*), which overlaps the 3' end of *pol* (Figure 1) [9,16,18,20].

The JSRV Gag polyprotein is cleaved into at least five products: matrix (MA), p15, capsid (CA), nucleocapsid (NC) and p4 [21]. At the N-terminus, MA contains a consensus myristilation signal that forms part of a putative M domain; mutations in this region alter the ability of JSRV to reach the cell membrane and release viral particles* in vitro* [21,22]. The late (L) domain, within the p15 protein, contains core amino acid motifs of PSAP and PPAY analogous to those present in M-PMV [23]. Consonant with other retroviruses, mutations in the JSRV L domain result in a defect of the late steps of viral replication cycle that impairs viral budding [21,24]. As described for other betaretroviruses, *pro* and *pol* are encoded in different open reading frames [9]. The *pro* gene expresses: (i) a deoxyuridine triphosphatase (dUTPase) that prevents incorporation of deoxyuridine triphosphate (dUTP) by the reverse transcriptase; and (ii) a protease (PR) that cleaves viral polyprotein precursors. The *pol* gene encodes the reverse transcriptase (RT) and the integrase (IN) involved in the reverse transcription and integration processes, respectively [18]. The Env glycoprotein is synthesized from a single spliced transcript of 2.4 kb that is cleaved into an *N*-terminal surface domain (SU) and *C*-terminal transmembrane domain (TM) (Figure 1) [20]. The first is exposed on the outer surface of the viral particle and mediates viral entry into the cells by interacting with Hyal2 (Hyaluronidase-2), the cellular receptor of JSRV [25]. The TM domain anchors JSRV to the cell lipid bilayer and confers the ability to induce cell transformation [26]. Unlike most retroviruses, whose TM domain harbors an immunosuppressive domain (ISD) that inhibits host immune function, JSRV TM seems to lack such sequence based on computational analyses [27,28]. However, the immunosuppressive properties of JSRV Env have not yet been assessed. The TM domain contains a short cytoplasmic tail of approximately 44 amino acid residues that bears a YXXM motif (Y for tyrosine, X for any amino acid and M for methionine) critical for transformation [26]. This is a distinctive feature from other retroviruses, which normally harbor YXXΦ motifs (Φ for any amino acid with a bulky hydrophobic chain) implicated in trafficking, endocytosis and pathogenesis [29]. Mutation analysis in the YXXM motif of JSRV Env indicates that the Y at position 590 is crucial for transformation [26,30,31,32]. In addition, JSRV-derived transformed cell lines show activation of Akt, an important PI3K downstream effector [26,31,33,34]. The role of Akt in JSRV Env transformation* in vivo* remains elusive since phosphorylation has not been observed by immunohistochemistry (IHC) of OPA lung sections [33]. However, Akt was found to be activated in ten out of twenty-seven samples of OPA lung tumors analyzed by western blotting [35].

Beside PI3K/Akt pathways, the Raf-MEK-MAPK signaling cascade is involved in JSRV-induced cell transformation, even though its role has not been completely elucidated. IHC assays revealed the presence of activated MEK1/2 and ERK1/2 in lung sections of naturally occurring and experimentally induced OPA [14,36]. Additionally, it was found that MEK1/2 and Ras inhibitors strongly reduced JSRV transformation of mouse NIH 3T3 fibroblasts in a dose dependent manner [37].

In summary, available evidence supports the idea that JSRV Env induces transformation at least in part by the activation of the Akt and Raf-MEK-MAPK pathways. The relevance of each signaling cascade may vary depending on the cell type analyzed, and it is likely that transformation is ultimately the result of the combinatory effects of these and other pathways. In addition, to date, it is not known how JSRV Env initiates the signaling cascades resulting in the activation of the Akt and Raf-MEK-MAPK pathways. The early steps in this process, including the mechanism employed by JSRV Env to engage the cell-signaling network, remain indeed the least understood area in the study of JSRV oncogenesis.

## 3. Ovine Pulmonary Adenocarcinoma

Sheep affected by OPA show different clinical signs, including progressive dyspnoea associated with loss of weight, and usually die for respiratory failure after a protracted incubation period in the naturally occurring cases [38]. One of the characteristic clinical signs of OPA is the production of copious amount of fluid in the lungs, which drains from the nostrils of affected sheep once their hind limbs are raised above their head. However, in many cases, no lung fluid can be observed and, therefore, definitive diagnosis of OPA can be made only after histopathological examination [10]. At post-mortem examination, naturally infected animals with advanced stages of OPA usually present a thin carcass with enlarged lungs infiltrated with tumor, and airways filled with fluid. Extrathoracic metastases have also been reported, but are generally rare [39]. Remarkably, JSRV infected animals do not develop antibodies against the virus, probably because of the expression of the highly related enJSRVs during ontogeny leading to immunological tolerance [25,40]. In natural conditions, JSRV can infect both adult sheep and lambs perhaps through aerosolized particles and maternal colostrum/milk [41,42]. Curiously, in naturally infected animals, JSRV is primarily detected in peripheral blood leucocytes and lymphoid organs rather than in lungs, and only a minority of naturally JSRV infected sheep develop OPA [42].

JSRV was found to infect and transform proliferating type 2 pneumocytes (also termed lung alveolar proliferating cells, LAPCs) in the lungs [43]. These cells are abundant in young lambs during postnatal development and adult sheep following injury to the bronchioalveolar epithelium. Indeed, it is possible to experimentally reproduce OPA only in young lambs after intratracheal infection with JSRV [43,44]. Interestingly, sheep with naturally occurring OPA often present a wide variety of other respiratory pathogens, including lungworms, Maedi-Visna virus (MVV, lentivirus), and other bacterial infections [45,46]. These pathogens may act synergistically to render sheep lungs more susceptible to JSRV transformation [42,43]. Similarly to most retroviruses, JSRV infects more efficiently cells in division, when the nuclear membrane dissolves [47]. Lung inflammation induced by primary pathogens may indeed promote active divisions of LAPCs to repair injured tissues, thus providing optimal conditions for JSRV transformation. This hypothesis is supported by the observation that chemically-induced lung lesions increase the number of LAPCs and render adult sheep susceptible to JSRV infection and OPA [43].

JSRV is unique among oncogenic viruses as it possesses a structural protein that functions as a dominant oncogene [13,14,15,32,48,49,50,51,52]. Hence, abundant viral replication leads to cell transformation, lung cancer and death of the animal hampering virus transmission. However, in natural settings, most JSRV infected animals do not develop any tumor and the virus is not detectable in lung cells. Virus and host have found a unique equilibrium where the target cells for JSRV transformation are rare and, as explained above, are found only in young lambs or adult sheep after lung injury. Hence, in most infected animals, JSRV targets cells of the lymphoreticular system where viral replication is very limited (indeed the virus is mainly detected in these cells by sensitive PCR assays [9,53]. In rare cases, when infection occurs in lambs or in animals with concomitant respiratory pathogens, JSRV targets and transform LAPCs, leading to OPA. The presence of lung fluid with abundant infectious JSRV particles in OPA affected animals may also facilitate the spread of the infection and the maintenance of the virus in the environment.

Goats are less susceptible to JSRV-induced pulmonary carcinogenesis. In nature, only a handful of reports have described OPA in this animal species at low incidence [54,55,56,57]. Experimentally, JSRV can also infect LAPCs of goat kids and lead to small tumor lesions [11,58]. However, available data suggest that goat cells are susceptible to viral infection but not to viral replication [11]. Interestingly, goats also harbor transcriptionally active endogenous retroviruses similar to enJSRVs that may lead to tolerance towards the exogenous JSRV [6,11].

## 4. Endogenous Sheep Betaretroviruses: enJSRVs

Sequence analyses conducted on a bacterial artificial chromosome (BAC) library derived from the genomic DNA of a single Texel ram revealed that the sheep genome harbors at least twenty-seven copies of endogenous betaretroviruses, highly related to the exogenous and pathogenic JSRV and hence termed enJSRVs [6]. These loci display the same genetic structure and organization of their exogenous counterpart; in particular, they share 85%–89% identity with *gag* and *env* of the infectious molecular clone JSRV_21_, and differ from the latter for the unique region at the 3' end (U3) as well as the three variable regions, VR1, VR2 and VR3 [6,9,59,60].

**Figure 2:**
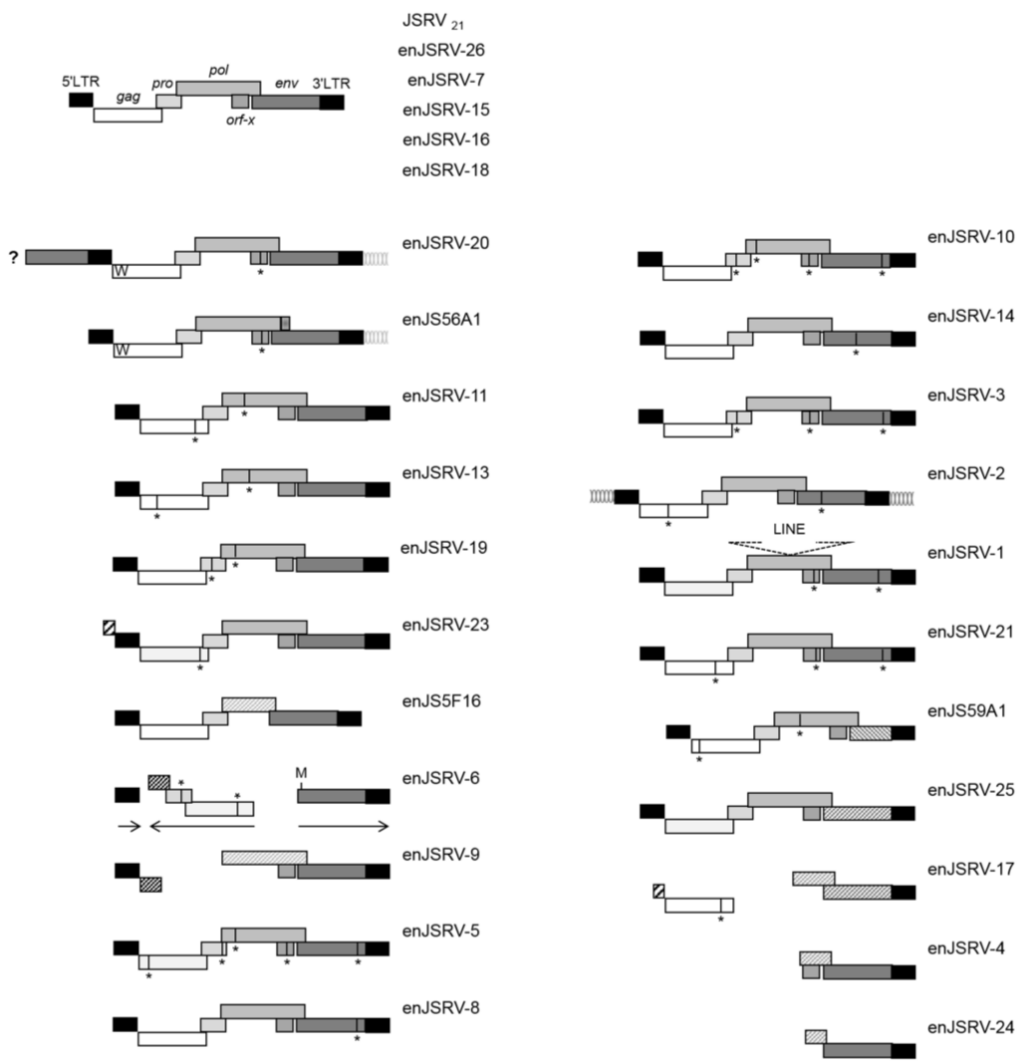
Genetic organization of enJSRV proviruses. All the genomic sequences flanking enJSRV proviruses display a six base pair duplication that is the hallmark of retroviral integration. The only exceptions are enJSRV-20, which contains a portion of an *env* gene before the 5' LTR (indicated by a dark grey box and ? mark), and enJSRV-2 that does not possess the same six base pairs sequences flanking its LTRs. At the top are shown the five enJSRVs displaying an intact genomic organization, typical of replication competent exogenous retroviruses. The two transdominant proviruses enJS56A1 and enJSRV-20 possess a tryptophan residue (W) at position 21 of Gag and identical 3' genomic flanking regions. The enJSRV-6 locus contains an additional methionine (M) in Env besides the canonical start codon present in JSRV and other enJSRV loci. In addition, in enJSRV-6, gag and pro are oriented in opposite direction compared to the 5' LTR, 3' LTR and env (indicated by horizontal arrows). enJSRV-1 possesses a long interspersed nucleotide element (LINE) within the pol coding region. Note that the following enJSRVs are insertionally polymorphic in the genome of domestic sheep: enJSRV-7, enJSRV-8, enJSRV-15, enJSRV-16, enJSRV-18, enJSRV-26 and enJS5F16. Premature termination codons are represented by a vertical line and an asterisk (*). Large deletions in proviral genomes are indicated by hatched boxes. enJSRV, endogenous Jaagsiekte sheep retrovirus; LTR, long terminal repeat (Figure modified from Arnaud *et al*. 2007 PLoS Pathogens) [6].

Most enJSRVs exhibit defective genomes with premature termination codons, large deletions and/or recombination. Sixteen loci possess intact open reading frames for *env*, even though two of them (enJSRV-4 and enJSRV-24) lack most of the other genes [6,59]. Five enJSRVs (enJSRV-7, enJSRV-15, enJSRV-16, enJSRV-18 and enJSRV-26) display an intact genomic organization and uninterrupted open reading frames for all the retroviral genes, similarly to the replication competent JSRV_21_ (Figure 2). In particular, enJSRV-15, enJSRV-16, enJSRV-18 and enJSRV-26 possess identical 5' and 3' LTRs, suggesting relatively recent integration into the host germ line. This hypothesis is further reinforced by the fact that two of these loci (enJSRV-16 and enJSRV-18) are 100% identical at the nucleotide level along their entire genomes [6]. Moreover, enJSRV-26, the youngest enJSRV isolated to date (less than 200 years ago) might even be the result of a unique integration event within the germ line of its host, as it was only recovered from the genome of a single Texel ram over several animals of different breads tested, including the DNA of five of its close relatives [6].

## 5. enJSRVs and Sheep Reproductive Biology

enJSRVs are abundantly expressed in sheep reproductive organs, including epithelia of the oviduct, cervix and uterus. enJSRV mRNAs can also be detected in lymphoid cells associated to the gut lamina propria, lung bronchial epithelial cells, and the cortico-medullary junction of the thymus, where T-lymphocytes undergo the process of maturation [43,53,59,61]. In the conceptus (embryo/fetus and associated extraembryonic membranes), enJSRVs *env* mRNA is detected in mononuclear trophoblast and is particularly abundant in trophoblast giant binucleate cells (BNCs) and multinucleated syncytia [59,61,62]. *In vivo*, loss-of-function experiments showed that enJSRVs Env favor conceptus growth and elongation and prevents differentiation of trophoblast giant BNCs, resulting in early pregnancy loss. These data indicate that enJSRV *env* mRNA is essential for conceptus elongation and trophectoderm growth in sheep [63]. Thus far,* in vitro* cell assays failed to demonstrate that enJSRVs Env display fusogenic properties; however, we cannot exclude that, following BNCs differentiation, they may also participate* in vivo* to the syncytiotrophoblast formation through cell fusions. Moreover, unlike other retroviral Env, enJSRVs Env do not harbor a canonical ISD sequence, though it remains still to be investigated whether or not they display an immunosuppressive activity and participate to the materno-fetal tolerance during pregnancy [27,28,64,65].

Recently, it was proposed that enJSRVs cell tropism within the reproductive tract might have been influenced by host restriction factors, such as bone marrow stromal cell antigen 2 (BST-2) [66,67,68]. BST-2, also known as tetherin, is a transmembrane protein that restricts many enveloped viruses, including retroviruses, filoviruses and herpesviruses [69,70,71,72]. It is expressed constitutively in several cell types, such as B cells, T cells, macrophages and cancer cell lines, and its expression can be induced by type I interferon (IFN) in response to viral infections [73,74,75]. In many ruminants, the developing conceptus secretes IFN tau (IFNT) that acts as a pregnancy recognition molecule to promote uterine implantation. Moreover, and similarly to other type I IFNs, IFNT activates signaling pathways involved in maintaining maternal tolerance of the fetal allograft and protecting the conceptus from viral infections [76]. During pregnancy, the steroid hormone progesterone is produced by the corpus luteum that acts on the endometrium to stimulate blastocyst growth and elongation to a filamentous conceptus [77]. In sheep, IFNT and progesterone enhance the expression of BST-2 and enJSRVs Env in the stroma and luminal endometrial epithelium, respectively [59,61,62,67]. Interestingly, in ruminants, the BST-2 gene is duplicated (referred to as oBST-2A and oBST-2B). Phylogenetic analyses indicate that this duplication occurred approximately 25 million years ago (MYA), before the speciation of the Bovinae subfamily and, thus, before the initial integration of enJSRVs in the sheep genome (approximately 5–7 MYA; more details in paragraph 7) [6,67,78]. *In vitro* experiments reveal that oBST-2A is able to block efficiently enJSRV particles release, whereas oBST2B impairs the normal cellular trafficking of JSRV envelope glycoproteins by sequestrating them within the Golgi apparatus. In turn, oBST2B leads to a reduction in Env incorporation into JSRV viral particles and a significant decrease in the viral infectivity [67,68]. Since the duplication of oBST-2 predated the initial invasion of enJSRVs in the sheep genome, it has been proposed that this gene might have been one of the selective forces that confined enJSRVs within specific areas of the reproductive tract, where these cellular restriction factors were not expressed at all, or at very low levels [67].

A recent study revealed that the enJSRVs expressed in sheep uterine endometrial epithelia can release viral particles into the uterine lumen [79]. By using a *trans*-species embryo model in which bovine embryos were transferred into ovine uteri, it was found that enJSRVs particles can potentially infect the trophoblast of the developing conceptus. Interestingly, the great majority of enJSRV sequences amplified from the endometria and uterine flushes of recipient ewes and transferred bovine embryos clustered mainly with the youngest and intact enJSRV loci, while older proviruses were rarely recovered from ovine endometria [79]. Any retroviral integration represents indeed a source of mutagenesis that, if uncontrolled, may jeopardize host survival. However, the absence of an ancestral and ubiquitously expressed enJSRV Env suggests that, unlike for other mammalian species, the capture of an *env* gene to fulfill a vital biological function (e.g., placental morphogenesis) has not occurred and that perhaps, *de novo* integrations of intact enJSRV loci in the sheep genome are necessary to render redundant the function provided by older proviruses.

## 6. enJSRVs and Host Defense

Besides their role in conceptus development, enJSRVs could protect their host against infections by related exogenous retroviruses. Expression of enJSRVs Env glycoproteins can block* in vitro* cell entry of related exogenous retroviruses by receptor interference. Standard entry assays revealed indeed that enJSRV Env mediates viral entry *via* Hyal2, which serves also as a cellular receptor for the exogenous JSRV and ENTV sheep betaretroviruses [25]. However, enJSRVs Env glycoproteins lack the YXXM motif critical for JSRV cell transformation and, therefore, are unable to induce foci in classical transformation assays of rodent and chicken cell lines [6,26].

Another example of host defense mechanism is offered by enJS56A1 that,* in vitro*, can interfere with the late replication steps of JSRV by a unique mechanism known as JLR, for “JSRV late restriction” [22]. The main determinant of JLR was mapped to the tryptophan residue at position 21 (W21) in enJS56A1 Gag, which substitutes an arginine (R) well conserved in betaretroviruses. The R21W mutation confers to enJS56A1 Gag a defective phenotype that is transdominant over JSRV as well as other enJSRVs [6,22]. In particular, it was demonstrated that enJS56A1 and JSRV Gag molecules seemingly form chimeric multimers that cannot traffic properly and undergo proteasomal degradation, thus impairing JSRV particles release [21,80]. Interestingly, sequence analyses unveiled that the chromosomal region containing the enJS56A1 locus has been amplified several times during sheep domestication, particularly in some breeds of *Ovis aries* (domestic sheep), reinforcing the hypothesis that this transdominant provirus might have provided an evolutionary advantage to its host [81]. The presence of a second transdominant provirus, enJSRV-20, that most likely arose by processes of recombination and/or gene conversion with enJS56A1, further supports the idea that sheep domestication has contributed to the selection and amplification of these proviruses [6,81]. Most interestingly, and unlike the other full-length and intact enJSRVs, enJSRV-26 recently evolved to escape JLR exerted by enJS56A1-like transdominant proviruses [6].

## 7. The Evolutionary History of enJSRVs and Their Hosts

Once retroviruses infect germ cells, they leave a permanent footprint in the genome of their hosts that can reveal the dynamics of host-pathogen interplay across long evolutionary periods. ERVs hold therefore great potential as informative markers for both determining the age of viruses, and gaining a better understanding of the gene flow between host and pathogen. Along this line, the characterization of enJSRVs distribution in various breeds of domestic sheep (*O. aries*) has provided valuable insights into the history of these viruses and the domestication of this animal species [82].

The “age” of a provirus can be inferred: (i) directly, by knowing the speciation time of phylogenetically related species; or (ii) indirectly, by assessing the sequence divergence between the 5' and 3' LTRs, and assuming that both regions were identical at the time of infection but evolved separately afterwards at the same evolutionary rate of noncoding regions. According to this, “young” ERVs possess identical or nearly identical LTRs, while “old” proviruses have significantly different LTRs. However, it is important to consider that 5' LTR and 3' LTR may have different evolutionary rates on each end of the locus, and that homologous ERVs may undergo diverse selective pressures depending on the species [83].

Sequence analyses and phylogenetic data indicate that enJSRVs entered the host genome approximately 5–7 million years ago (MYA), before speciation of *Ovis* and *Capra* genera [6]. Some enJSRV loci were recovered in all the species belonging to the *Ovis* genus, such as *O. aries*, *O. nivicola*, *O. canadensis* and *O. dalli*, whereas others were restricted only to domestic sheep, including eight insertionally polymorphic enJSRVs. These findings and the current knowledge on ruminant evolution suggest that the insertionally polymorphic enJSRVs entered the sheep genome less than 9000 years ago, after sheep domestication [6].

enJS56A1 was positively selected during sheep domestication, most probably as it conferred advantages in protecting the host against infections by related exogenous retroviruses. Interestingly, enJSRV-20 was also found to possess the same R21W mutation in Gag that confers the defective and transdominant phenotype to enJS56A1 [6]. enJS56A1 and enJSRV-20 are 99% identical at the nucleotide level and contain intact open reading frames for all of the retroviral genes but *orf-x*. In particular, the first possesses a two base pairs deletion in *pol* that causes a frameshift and yields a shorter protein than enJSRV-20 and the exogenous JSRV_21_ Pol. In addition, both enJS56A1 and enJSRV-20 share identical 3' genomic flanking regions, even though the latter contains a portion of an *env* gene immediately before the 5' LTR (Figure 2) [6]. Overall, these findings suggest that enJSRV-20 arose from various processes of recombination between enJS56A1 and other proviruses, rather than independent mutations.

It has been estimated that enJS56A1 and enJSRV-20 entered the sheep genome within the last 3 MYA, during *Ovis* speciation. The exogenous enJSRV-like virus from which these transdominant proviruses derived possessed the “wild-type” R residue at position 21 in Gag when it first entered host genome, in order to replicate and infect the host germ line. Only subsequently, around the time of sheep domestication, the “transdominant” enJS56A1 genotype harboring W21 appeared in the host genome and became fixed in the host population [6].

During evolution, transdominant proviruses might have been positively selected for their ability to interfere with related exogenous pathogenic retroviruses and enJSRVs already colonizing sheep genome [6]. One can reason that, with domestication, a relatively large number of animals were suddenly kept in restricted spaces, and this likely facilitated the spread of infectious agents more easily than before: under these circumstances, sheep with transdominant proviruses might have had a selective advantage over animals not harboring them [6,80,81]. In addition, a recent study demonstrated that the chromosomal location containing enJS56A1 has been amplified several times, especially in some breeds of domestic sheep, further supporting the idea that sheep domestication has contributed to the selection and amplification of transdominant proviruses [6]. Along the same line, one may speculate that animals infected with JSRV and that develop lung tumors may possess a lower copy number variation of transdominant enJS56A1-like proviruses compared to those that do not develop OPA, even though thus far not enough studies have been conducted to firmly address this point.

## 8. It Takes All the Running You Can Do, to Keep in the Same Place

For many years, ERVs have been considered as merely molecular “junk” or parasites. It is now clear that host genomes have coevolved with ERVs, preventing or minimizing the deleterious consequences of their unrestrained integrations, while capitalizing on their adaptive potential: in other words, turning some “junk” into treasure [84]. Indeed, over time, some ERVs have been positively and repeatedly selected and fulfill now useful functions in diverse aspects of host biology, including antiviral activity and placental morphogenesis [65,85,86,87,88]. An example of such coevolution is represented by sheep and enJSRVs that, over time, have engaged in mutualistic relationships in which both of them have gained reciprocal benefits: sheep have contributed to the maintenance of enJSRVs by transmission to subsequent generations, while enJSRVs have played critical roles in host survival.

enJSRVs are critical for successful sheep pregnancy as, in absence of functional Envs, ewes abort at the early stages of gestation [63,89]. However, several lines of evidences suggest that enJSRVs were not positively selected and fixed in the sheep population for their role in placentation. Indeed, mammalian placenta predates the first enJSRVs integration in the genome of the Caprinae, so it is possible to speculate that enJSRVs might have been initially co-opted for their ability to protect their host against infection by related pathogenic retroviruses. This notion is further reinforced by the presence of at least two transdominant proviruses (enJS56A1 and enJSRV-20) in the sheep genome, and the amplification of their chromosomal location in domestic breeds [6,81].

**Figure 3:**
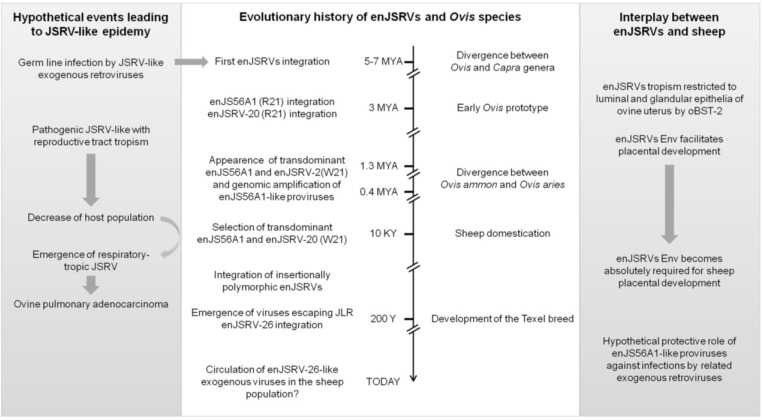
Hypothetical adaptation and counter-adaptation events between enJSRVs, JSRV and their host during evolution. See text for more details. enJSRV, endogenous Jaagsiekte sheep retrovirus; JLR, JSRV late restriction; JSRV, Jaagsiekte sheep retrovirus; oBST-2, ovine bone marrow stromal cell antigen 2; R21, arginine residue at position 21 of enJS56A1/enJSRV-20 Gag; W21, tryptophan residue at position 21 of enJS56A1/enJSRV-20 Gag (Figure modified from Varela et al. 2009) [65].

If one were to reconstruct the evolutionary interplay between JSRV, enJSRVs and domestic sheep, one should start by assuming that enJSRVs arose from the germ line infection of a JSRV-like exogenous retrovirus that, most likely, had a reproductive tract tropism (like enJSRVs today). Next, natural selection might have exerted positive pressure on transdominant proviruses able to protect their host and this, in turn, might have “forced” exogenous viruses to “run-away”, by acquiring different tissue tropism. Most likely, this represents the strategy adopted by JSRV to avoid JLR: replicating in tissues where interfering enJSRVs are not highly expressed, such as the lung bronchialveolar epithelial cells [43,53,59,61,62]. At the same time, the emergence of animals harboring transdominant proviruses must have exerted selective pressure for the appearance of viruses able to escape JLR such as enJSRV-26, the “youngest” enJSRV isolated to date (Figure 3) [6].

**Figure 4:**
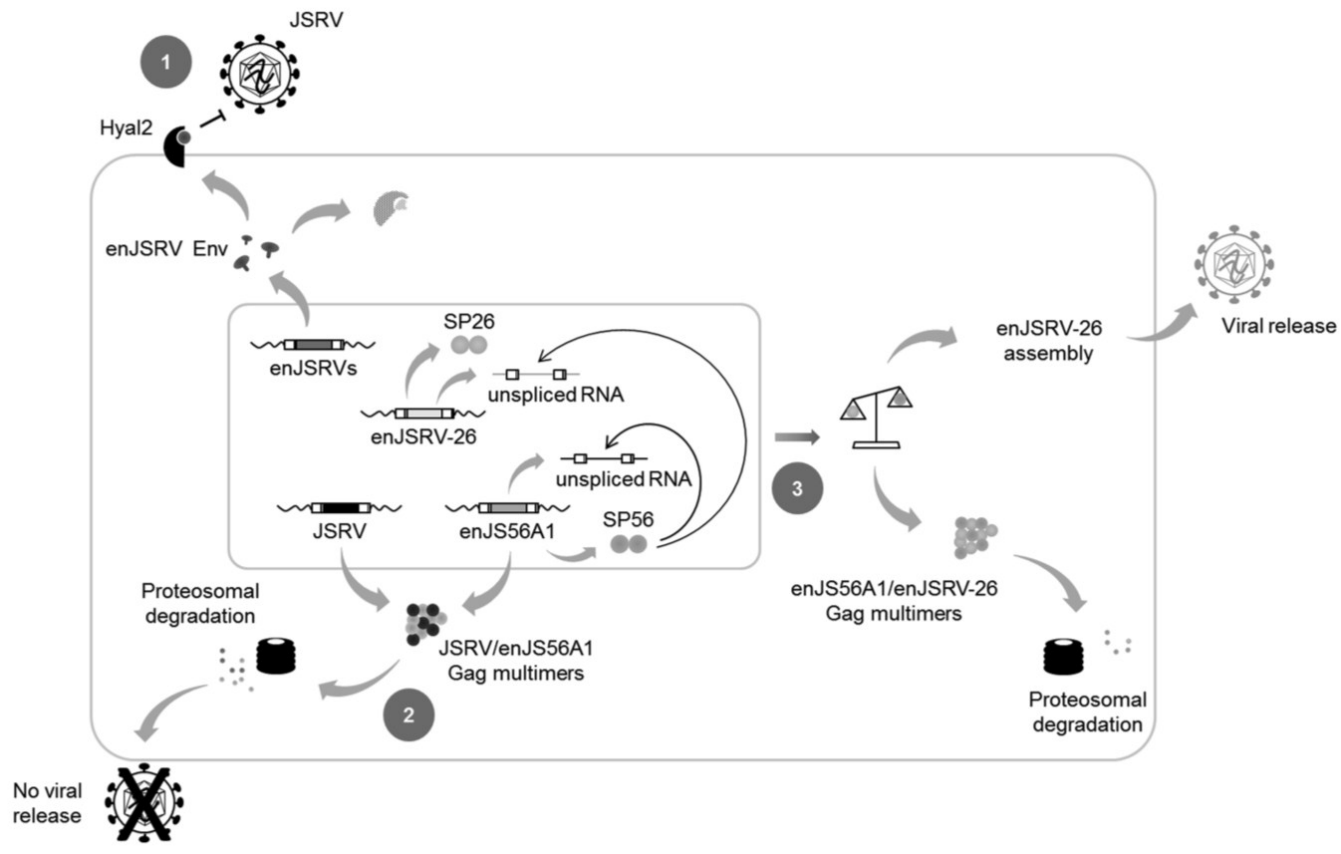
Mechanisms of restriction induced by enJSRVs, and JLR escape. enJSRVs can inhibit JSRV entry at the cell entry by receptor interference, as both endogenous and exogenous retroviruses use Hyal2 as cellular receptor. The binding between enJSRVs Env and Hyal2 (either at the plasma membrane or in the cytoplasm) decreases the availability of the latter at the cell surface, thereby inhibiting JSRV entry into target cells (1); In addition, some enJSRVs, such as enJS56A1, can block JSRV at post-integration steps of viral cycle. This mechanism of interference, known as JLR, most likely impairs viral particle transport or exit, and is exerted by transdominant Gag proteins: these form aggregates with JSRV Gag that are subsequently targeted to the proteasome where they are degraded (2); The ability of enJSRV-26 to elude JLR restriction is dependent on the impaired function of its SP. Consequently, the enJSRV-26 and enJS56A1 unspliced RNAs compete for the only functional SP (*i.e.*, SP56), resulting in a reduced expression of transdominant Gag. Thus an unbalanced ratio between enJSRV-26 and enJS56A1 Gag polyproteins is established leading, at the same time, to a partial degradation and particle release of the first (3). enJSRV endogenous Jaagsiekte sheep retrovirus; Hyal2 hyaluronidase2; JSRV Jaagsiekte sheep retrovirus.

The main determinant of JLR escape is an aspartic acid at position 6 (D6) of the signal peptide (SP) of enJSRV-26 envelope glycoprotein (SP26) [81]. This amino acid residue substitutes an alanine (A) well conserved in the exogenous and pathogenic JSRV as well as in all enJSRVs. Interestingly, it has been demonstrated that, similarly to MMTV SP, JSRV/enJSRV SP plays a critical role in viral replication cycle, as it enhances Gag protein synthesis and particle release [90,91,92,93]. The A6D substitution was found to be responsible for altering SP26 intracellular localization as well as its function as a post-transcriptional regulator of viral gene expression. In particular, confocal microscopy analysis revealed that SP26 displays a diffuse staining within the nucleus and the cytoplasm of transfected cells with no accumulation in the nucleolus, most likely due to the presence of a hydrophilic residue, such as aspartic acid, that may alter the overall conformation of the protein [90,91,92,93]. Several studies demonstrated that many DNA and RNA-virus proteins localize to the nucleus to take over host cell functions and favor viral replication [94]. It is therefore possible to hypothesize that the inability of SP26 to reach the nucleolus may affect its ability to participate in the nucleocytoplasmic trafficking of viral unspliced RNAs, thereby decreasing the synthesis of enJSRV-26 Gag proteins. Interestingly, interference assays demonstrated that enJSRV-26 mostly relies on the presence of the functional SP of enJS56A1 envelope protein (SP56) for the synthesis of its Gag polyproteins. In addition, western blot analyses indicated that enJSRV-26 is not totally exempted from JLR since a certain amount of its Gag protein is restricted by enJS56A1, suggesting that the ratio between enJSRV-26 and enJS56A1 Gag is critical to elude restriction. Overall, these findings suggest that JLR escape depends on the stoichiometry between enJS56A1 and enJSRV-26 Gag which, in turn, is regulated by the SPs of these viruses (Figure 4) [81].

The presence of such escape provirus strongly supports the hypothesis that transdominant enJSRVs might have played a critical role as restriction factors against related exogenous retroviruses. The fact that: (i) enJSRV-26 was detected only in a single Texel ram; and (ii) enJSRV26-like sequences were found in the luminal endometrial epithelium of two Texel animals strongly argues that an exogenous retrovirus related to enJSRV-26 is still circulating within the sheep population [6,79]. Moreover, it is noteworthy that five of the twenty-seven enJSRV loci isolated to date (enJSRV-7, enJSRV-15, enJSRV-16, enJSRV-18 and enJSRV-26) are insertionally polymorphic and display an intact genomic organization [6]. Overall these findings suggest that, in sheep, the process of endogenization is still ongoing and, therefore, the evolutionary interplay between endogenous and exogenous sheep betaretroviruses and their hosts has not yet reached an equilibrium.

Host-pathogen interaction is modeled as a typical “arms race”, in which each partner gains advantage over the other by maximizing its own fitness at the other expenses. Co-evolutionary processes favor rapid rates of evolution that lead to constant natural selection for adaptation and counter-adaptation. This “back-and-forth” interplay has been highly dynamic and contributed to rapid changes in viral and host strategies, with each “species” rushing to evolve the upper hand in the interaction in a never ending struggle. Along this line, JSRV, enJSRVs and domestic sheep represent an interesting model to study the evolutionary interplay between host and pathogen.

## References

[1] Stoye J.P. (2012). Studies of endogenous retroviruses reveal a continuing evolutionary saga. Nat. Rev. Microbiol..

[2] Jern P., Coffin J.M. (2008). Effects of retroviruses on host genome function. Annu. Rev. Genet..

[3] Gifford R., Tristem M. (2003). The evolution, distribution and diversity of endogenous retroviruses. Virus Genes.

[4] Jung W.Y., Yu S.L., Seo D.W., Jung K.C., Cho I.C., Lim H.T., Jin D.I., Lee J.H. (2012). Characterization of insertional variation of porcine endogenous retroviruses in six different pig breeds. Asian-Australas. J. Anim. Sci..

[5] Kimsa M.C., Strzalka-Mrozik B., Kimsa M.W., Gola J., Nicholson P., Lopata K., Mazurek U. (2014). Porcine endogenous retroviruses in xenotransplantation--molecular aspects. Viruses.

[6] Arnaud F., Caporale M., Varela M., Biek R., Chessa B., Alberti A., Golder M., Mura M., Zhang Y.P., Yu L. (2007). A paradigm for virus-host coevolution: Sequential counter-adaptations between endogenous and exogenous retroviruses. PLoS Pathog..

[7] Elleder D., Kim O., Padhi A., Bankert J.G., Simeonov I., Schuster S.C., Wittekindt N.E., Motameny S., Poss M. (2012). Polymorphic integrations of an endogenous gammaretrovirus in the mule deer genome. J. Virol..

[8] Tarlinton R.E., Meers J., Young P.R. (2006). Retroviral invasion of the koala genome. Nature.

[9] Palmarini M., Sharp J.M., de las Heras M., Fan H. (1999). Jaagsiekte sheep retrovirus is necessary and sufficient to induce a contagious lung cancer in sheep. J. Virol..

[10] De las Heras M., Gonzalez L., Sharp J.M. (2003). Pathology of ovine pulmonary adenocarcinoma. Curr. Top. Microbiol. Immunol..

[11] Caporale M., Martineau H., de las Heras M., Murgia C., Huang R., Centorame P., di Francesco G., di Gialleonardo L., Spencer T.E., Griffiths D.J. (2013). Host species barriers to Jaagsiekte sheep retrovirus replication and carcinogenesis. J. Virol..

[12] Palmarini M., Fan H. (2001). Retrovirus-induced ovine pulmonary adenocarcinoma, an animal model for lung cancer. J. Natl. Cancer Inst..

[13] Liu S.L., Miller A.D. (2007). Oncogenic transformation by the jaagsiekte sheep retrovirus envelope protein. Oncogene.

[14] Caporale M., Cousens C., Centorame P., Pinoni C., De las Heras M., Palmarini M. (2006). Expression of the jaagsiekte sheep retrovirus envelope glycoprotein is sufficient to induce lung tumors in sheep. J. Virol..

[15] Wootton S.K., Halbert C.L., Miller A.D. (2005). Sheep retrovirus structural protein induces lung tumours. Nature.

[16] York D.F., Vigne R., Verwoerd D.W., Querat G. (1992). Nucleotide sequence of the jaagsiekte retrovirus, an exogenous and endogenous type D and B retrovirus of sheep and goats. J. Virol..

[17] York D.F., Vigne R., Verwoerd D.W., Querat G. (1991). Isolation, identification, and partial cDNA cloning of genomic RNA of jaagsiekte retrovirus, the etiological agent of sheep pulmonary adenomatosis. J. Virol..

[18] Palmarini M., Fan H. (2003). Molecular biology of jaagsiekte sheep retrovirus. Curr. Top. Microbiol. Immunol..

[19] DeMartini J.C., Bishop J.V., Allen T.E., Jassim F.A., Sharp J.M., de las Heras M., Voelker D.R., Carlson J.O. (2001). Jaagsiekte sheep retrovirus proviral clone JSRV(JS7), derived from the JS7 lung tumor cell line, induces ovine pulmonary carcinoma and is integrated into the surfactant protein A gene. J. Virol..

[20] Palmarini M., Murgia C., Fan H. (2002). Spliced and prematurely polyadenylated Jaagsiekte sheep retrovirus-specific RNAs from infected or transfected cells. Virology.

[21] Murcia P.R., Arnaud F., Palmarini M. (2007). The transdominant endogenous retrovirus enJS56A1 associates with and blocks intracellular trafficking of Jaagsiekte sheep retrovirus Gag. J. Virol..

[22] Mura M., Murcia P., Caporale M., Spencer T.E., Nagashima K., Rein A., Palmarini M. (2004). Late viral interference induced by transdominant Gag of an endogenous retrovirus. Proc. Natl. Acad. Sci. USA.

[23] Gottwein E., Bodem J., Muller B., Schmechel A., Zentgraf H., Krausslich H.G. (2003). The Mason-Pfizer monkey virus PPPY and PSAP motifs both contribute to virus release. J. Virol..

[24] Freed E.O. (2002). Viral late domains. J. Virol..

[25] Spencer T.E., Mura M., Gray C.A., Griebel P.J., Palmarini M. (2003). Receptor usage and fetal expression of ovine endogenous betaretroviruses: Implications for coevolution of endogenous and exogenous retroviruses. J. Virol..

[26] Palmarini M., Maeda N., Murgia C., De-Fraja C., Hofacre A., Fan H. (2001). A phosphatidylinositol 3-kinase docking site in the cytoplasmic tail of the Jaagsiekte sheep retrovirus transmembrane protein is essential for envelope-induced transformation of NIH 3T3 cells. J. Virol..

[27] Cianciolo G.J., Copeland T.D., Oroszlan S., Snyderman R. (1985). Inhibition of lymphocyte proliferation by a synthetic peptide homologous to retroviral envelope proteins. Science.

[28] Henzy J.E., Coffin J.M. (2013). Betaretroviral envelope subunits are noncovalently associated and restricted to the mammalian class. J. Virol..

[29] Ye L., Bu Z., Vzorov A., Taylor D., Compans R.W., Yang C. (2004). Surface stability and immunogenicity of the human immunodeficiency virus envelope glycoprotein: Role of the cytoplasmic domain. J. Virol..

[30] Liu S.L., Miller A.D. (2005). Transformation of madin-darby canine kidney epithelial cells by sheep retrovirus envelope proteins. J. Virol..

[31] Liu S.L., Lerman M.I., Miller A.D. (2003). Putative phosphatidylinositol 3-kinase (PI3K) binding motifs in ovine betaretrovirus Env proteins are not essential for rodent fibroblast transformation and PI3K/Akt activation. J. Virol..

[32] Allen T.E., Sherrill K.J., Crispell S.M., Perrott M.R., Carlson J.O., DeMartini J.C. (2002). The jaagsiekte sheep retrovirus envelope gene induces transformation of the avian fibroblast cell line DF-1 but does not require a conserved SH2 binding domain. J. Gen. Virol..

[33] Zavala G., Pretto C., Chow Y.H., Jones L., Alberti A., Grego E., De las Heras M., Palmarini M. (2003). Relevance of Akt phosphorylation in cell transformation induced by Jaagsiekte sheep retrovirus. Virology.

[34] Varela M., Chow Y.H., Sturkie C., Murcia P., Palmarini M. (2006). Association of RON tyrosine kinase with the Jaagsiekte sheep retrovirus envelope glycoprotein. Virology.

[35] Suau F., Cottin V., Archer F., Croze S., Chastang J., Cordier G., Thivolet-Bejui F., Mornex J.F., Leroux C. (2006). Telomerase activation in a model of lung adenocarcinoma. Eur. Respir. J..

[36] De Las Heras M., Ortin A., Benito A., Summers C., Ferrer L.M., Sharp J.M. (2006). In-situ demonstration of mitogen-activated protein kinase Erk 1/2 signalling pathway in contagious respiratory tumours of sheep and goats. J. Comp. Pathol..

[37] Maeda N., Fu W., Ortin A., de las Heras M., Fan H. (2005). Roles of the Ras-MEK-mitogen-activated protein kinase and phosphatidylinositol 3-kinase-Akt-mTOR pathways in Jaagsiekte sheep retrovirus-induced transformation of rodent fibroblast and epithelial cell lines. J. Virol..

[38] Sharp J.M., DeMartini J.C. (2003). Natural history of JSRV in sheep. Curr. Top. Microbiol. Immunol..

[39] Demartini J.C., Rosadio R.H., Lairmore M.D. (1988). The etiology and pathogenesis of ovine pulmonary carcinoma (sheep pulmonary adenomatosis). Vet. Microbiol..

[40] Ortin A., Minguijon E., Dewar P., Garcia M., Ferrer L.M., Palmarini M., Gonzalez L., Sharp J.M., de las Heras M. (1998). Lack of a specific immune response against a recombinant capsid protein of Jaagsiekte sheep retrovirus in sheep and goats naturally affected by enzootic nasal tumour or sheep pulmonary adenomatosis. Vet. Immunol. Immunopathol..

[41] Grego E., de Meneghi D., Alvarez V., Benito A.A., Minguijon E., Ortin A., Mattoni M., Moreno B., Perez de Villarreal M., Alberti A. (2008). Colostrum and milk can transmit jaagsiekte retrovirus to lambs. Vet. Microbiol..

[42] Caporale M., Centorame P., Giovannini A., Sacchini F., di Ventura M., de las Heras M., Palmarini M. (2005). Infection of lung epithelial cells and induction of pulmonary adenocarcinoma is not the most common outcome of naturally occurring JSRV infection during the commercial lifespan of sheep. Virology.

[43] Murgia C., Caporale M., Ceesay O., Di Francesco G., Ferri N., Varasano V., de las Heras M., Palmarini M. (2011). Lung adenocarcinoma originates from retrovirus infection of proliferating type 2 pneumocytes during pulmonary post-natal development or tissue repair. PLoS Pathog..

[44] Sharp J.M., Herring A.J. (1983). Sheep pulmonary adenomatosis: Demonstration of a protein which cross-reacts with the major core proteins of Mason-Pfizer monkey virus and mouse mammary tumour virus. J. Gen. Virol..

[45] Snyder S.P., DeMartini J.C., Ameghino E., Caletti E. (1983). Coexistence of pulmonary adenomatosis and progressive pneumonia in sheep in the central sierra of Peru. Am. J. Vet. Res..

[46] Dawson M., Done S.H., Venables C., Jenkins C.E. (1990). Maedi-visna and sheep pulmonary adenomatosis: A study of concurrent infection. Br. Vet. J..

[47] Suzuki Y., Craigie R. (2007). The road to chromatin-nuclear entry of retroviruses. Nat. Rev. Microbiol..

[48] Alberti A., Murgia C., Liu S.L., Mura M., Cousens C., Sharp M., Miller A.D., Palmarini M. (2002). Envelope-induced cell transformation by ovine betaretroviruses. J. Virol..

[49] Cousens C., Maeda N., Murgia C., Dagleish M.P., Palmarini M., Fan H. (2007). *In vivo* tumorigenesis by Jaagsiekte sheep retrovirus (JSRV) requires Y590 in Env TM, but not full-length orfX open reading frame. Virology.

[50] Maeda N., Palmarini M., Murgia C., Fan H. (2001). Direct transformation of rodent fibroblasts by jaagsiekte sheep retrovirus DNA. Proc. Natl. Acad. Sci. USA.

[51] Rai S.K., Duh F.M., Vigdorovich V., Danilkovitch-Miagkova A., Lerman M.I., Miller A.D. (2001). Candidate tumor suppressor HYAL2 is a glycosylphosphatidylinositol (GPI)-anchored cell-surface receptor for jaagsiekte sheep retrovirus, the envelope protein of which mediates oncogenic transformation. Proc. Natl. Acad. Sci. USA.

[52] Maeda N., Fan H. (2008). Signal transduction pathways utilized by enzootic nasal tumor virus (ENTV-1) envelope protein in transformation of rat epithelial cells resemble those used by jaagsiekte sheep retrovirus. Virus Genes.

[53] Palmarini M., Holland M.J., Cousens C., Dalziel R.G., Sharp J.M. (1996). Jaagsiekte retrovirus establishes a disseminated infection of the lymphoid tissues of sheep affected by pulmonary adenomatosis. J. Gen. Virol..

[54] Rajya B.S., Singh C.M. (1964). The Pathology of Pneumonia and Associated Respiratory Disease of Sheep and Goats. I. Occurrence of jagziekte and maedi in sheep and goats in India. Am. J. Vet. Res..

[55] Nobel T.A. (1958). Pulmonary adenomatosies (jaagsiekte) in sheep with special reference to its occurrence in Israel. Refu. Vet. (Israel).

[56] Banerjee M., Gupta P.P. (1979). Note on maedi and jaagsiekte in sheep and goats in Ludhiana area of Punjab. Indian J. Anim. Sci..

[57] Stamp J.T., Nisbet D.I. (1963). Pneumonia of sheep. J. Comp. Pathol..

[58] Summers C., Benito A., Ortin A., Garcia de Jalon J.A., Gonzalez L., Norval M., Sharp J.M., de las Heras M. (2012). The distribution of immune cells in the lungs of classical and atypical ovine pulmonary adenocarcinoma. Vet. Immunol. Immunopathol..

[59] Palmarini M., Hallwirth C., York D., Murgia C., de Oliveira T., Spencer T., Fan H. (2000). Molecular cloning and functional analysis of three type D endogenous retroviruses of sheep reveal a different cell tropism from that of the highly related exogenous jaagsiekte sheep retrovirus. J. Virol..

[60] Bai J., Zhu R.Y., Stedman K., Cousens C., Carlson J., Sharp J.M., DeMartini J.C. (1996). Unique long terminal repeat U3 sequences distinguish exogenous jaagsiekte sheep retroviruses associated with ovine pulmonary carcinoma from endogenous loci in the sheep genome. J. Virol..

[61] Spencer T.E., Stagg A.G., Joyce M.M., Jenster G., Wood C.G., Bazer F.W., Wiley A.A., Bartol F.F. (1999). Discovery and characterization of endometrial epithelial messenger ribonucleic acids using the ovine uterine gland knockout model. Endocrinology.

[62] Palmarini M., Gray C.A., Carpenter K., Fan H., Bazer F.W., Spencer T.E. (2001). Expression of endogenous betaretroviruses in the ovine uterus: Effects of neonatal age, estrous cycle, pregnancy, and progesterone. J. Virol..

[63] Dunlap K.A., Palmarini M., Varela M., Burghardt R.C., Hayashi K., Farmer J.L., Spencer T.E. (2006). Endogenous retroviruses regulate periimplantation placental growth and differentiation. Proc. Natl. Acad. Sci. USA.

[64] Mangeney M., Renard M., Schlecht-Louf G., Bouallaga I., Heidmann O., Letzelter C., Richaud A., Ducos B., Heidmann T. (2007). Placental syncytins: Genetic disjunction between the fusogenic and immunosuppressive activity of retroviral envelope proteins. Proc. Natl. Acad. Sci. USA.

[65] Varela M., Spencer T.E., Palmarini M., Arnaud F. (2009). Friendly viruses: The special relationship between endogenous retroviruses and their host. Ann. NY Acad. Sci..

[66] Black S.G., Arnaud F., Palmarini M., Spencer T.E. (2010). Endogenous retroviruses in trophoblast differentiation and placental development. Am. J. Reprod. Immunol..

[67] Arnaud F., Black S.G., Murphy L., Griffiths D.J., Neil S.J., Spencer T.E., Palmarini M. (2010). Interplay between ovine bone marrow stromal cell antigen 2/tetherin and endogenous retroviruses. J. Virol..

[68] Murphy L., Varela M., Desloire S., Ftaich N., Murgia C., Golder M., Neil S., Spencer T.E., Wootton S.K., Lavillette D. (2014). The sheep tetherin paralog, oBST2B, blocks envelope glycoprotein incorporation into nascent retroviral virions. J. Virol..

[69] Jouvenet N., Neil S.J., Zhadina M., Zang T., Kratovac Z., Lee Y., McNatt M., Hatziioannou T., Bieniasz P.D. (2009). Broad-spectrum inhibition of retroviral and filoviral particle release by tetherin. J. Virol..

[70] Kaletsky R.L., Francica J.R., Agrawal-Gamse C., Bates P. (2009). Tetherin-mediated restriction of filovirus budding is antagonized by the Ebola glycoprotein. Proc. Natl. Acad. Sci. USA.

[71] Mansouri M., Viswanathan K., Douglas J.L., Hines J., Gustin J., Moses A.V., Fruh K. (2009). Molecular mechanism of BST2/tetherin downregulation by K5/MIR2 of Kaposi’s sarcoma-associated herpesvirus. J. Virol..

[72] Sakuma T., Noda T., Urata S., Kawaoka Y., Yasuda J. (2009). Inhibition of Lassa and Marburg virus production by tetherin. J. Virol..

[73] Yan N., Chen Z.J. (2012). Intrinsic antiviral immunity. Nat. Immunol..

[74] Van Damme N., Goff D., Katsura C., Jorgenson R.L., Mitchell R., Johnson M.C., Stephens E.B., Guatelli J. (2008). The interferon-induced protein BST-2 restricts HIV-1 release and is downregulated from the cell surface by the viral Vpu protein. Cell Host Microbe.

[75] Neil S.J., Zang T., Bieniasz P.D. (2008). Tetherin inhibits retrovirus release and is antagonized by HIV-1 Vpu. Nature.

[76] Bazer F.W., Burghardt R.C., Johnson G.A., Spencer T.E., Wu G. (2008). Interferons and progesterone for establishment and maintenance of pregnancy: Interactions among novel cell signaling pathways. Reprod. Biol..

[77] Spencer T.E., Johnson G.A., Bazer F.W., Burghardt R.C., Palmarini M. (2007). Pregnancy recognition and conceptus implantation in domestic ruminants: Roles of progesterone, interferons and endogenous retroviruses. Reprod. Fertil. Dev..

[78] Hassanin A., Douzery E.J. (2003). Molecular and morphological phylogenies of ruminantia and the alternative position of the moschidae. Syst. Biol..

[79] Black S.G., Arnaud F., Burghardt R.C., Satterfield M.C., Fleming J.A., Long C.R., Hanna C., Murphy L., Biek R., Palmarini M. (2010). Viral particles of endogenous betaretroviruses are released in the sheep uterus and infect the conceptus trophectoderm in a transspecies embryo transfer model. J. Virol..

[80] Arnaud F., Murcia P.R., Palmarini M. (2007). Mechanisms of late restriction induced by an endogenous retrovirus. J. Virol..

[81] Armezzani A., Arnaud F., Caporale M., di Meo G., Iannuzzi L., Murgia C., Palmarini M. (2011). The signal peptide of a recently integrated endogenous sheep betaretrovirus envelope plays a major role in eluding gag-mediated late restriction. J. Virol..

[82] Chessa B., Pereira F., Arnaud F., Amorim A., Goyache F., Mainland I., Kao R.R., Pemberton J.M., Beraldi D., Stear M.J. (2009). Revealing the history of sheep domestication using retrovirus integrations. Science.

[83] Martins H., Villesen P. (2011). Improved integration time estimation of endogenous retroviruses with phylogenetic data. PLoS One.

[84] Goodier J.L., Kazazian H.H. (2008). Retrotransposons revisited: The restraint and rehabilitation of parasites. Cell.

[85] Redelsperger F., Cornelis G., Vernochet C., Tennant B.C., Catzeflis F., Mulot B., Heidmann O., Heidmann T., Dupressoir A. (2014). Capture of syncytin-Mar1, a fusogenic endogenous retroviral envelope gene involved in placentation in the Rodentia squirrel-related clade. J. Virol..

[86] Dupressoir A., Marceau G., Vernochet C., Benit L., Kanellopoulos C., Sapin V., Heidmann T. (2005). Syncytin-A and syncytin-B, two fusogenic placenta-specific murine envelope genes of retroviral origin conserved in Muridae. Proc. Natl. Acad. Sci. USA.

[87] Cornelis G., Heidmann O., Degrelle S.A., Vernochet C., Lavialle C., Letzelter C., Bernard-Stoecklin S., Hassanin A., Mulot B., Guillomot M. (2013). Captured retroviral envelope syncytin gene associated with the unique placental structure of higher ruminants. Proc. Natl. Acad. Sci. USA.

[88] Blaise S., de Parseval N., Benit L., Heidmann T. (2003). Genomewide screening for fusogenic human endogenous retrovirus envelopes identifies syncytin 2, a gene conserved on primate evolution. Proc. Natl. Acad. Sci. USA.

[89] Dunlap K.A., Palmarini M., Spencer T.E. (2006). Ovine endogenous betaretroviruses (enJSRVs) and placental morphogenesis. Placenta.

[90] Nitta T., Hofacre A., Hull S., Fan H. (2009). Identification and mutational analysis of a Rej response element in Jaagsiekte sheep retrovirus RNA. J. Virol..

[91] Hofacre A., Nitta T., Fan H. (2009). Jaagsiekte sheep retrovirus encodes a regulatory factor, Rej, required for synthesis of Gag protein. J. Virol..

[92] Caporale M., Arnaud F., Mura M., Golder M., Murgia C., Palmarini M. (2009). The signal peptide of a simple retrovirus envelope functions as a posttranscriptional regulator of viral gene expression. J. Virol..

[93] Byun H., Halani N., Mertz J.A., Ali A.F., Lozano M.M., Dudley J.P. (2010). Retroviral Rem protein requires processing by signal peptidase and retrotranslocation for nuclear function. Proc. Natl. Acad. Sci. USA.

[94] Wang L., Ren X.M., Xing J.J., Zheng A.C. (2010). The nucleolus and viral infection. Virol. Sin..

